# Supplementary material for: Effect of acupuncture or moxibustion at Acupoints Weizhong (BL40) or Chize (LU5) on the change in lumbar temperature in healthy adults: A study protocol for a randomized controlled trial with a 2 × 2 factorial design
Source: PLoS One. 2023 Oct 30;18(10):e0291536. doi: 10.1371/journal.pone.0291536 (PMC10615297; doi:10.1371/journal.pone.0291536)
Supplement: S1 File — (DOCX) [file pone.0291536.s002.docx]

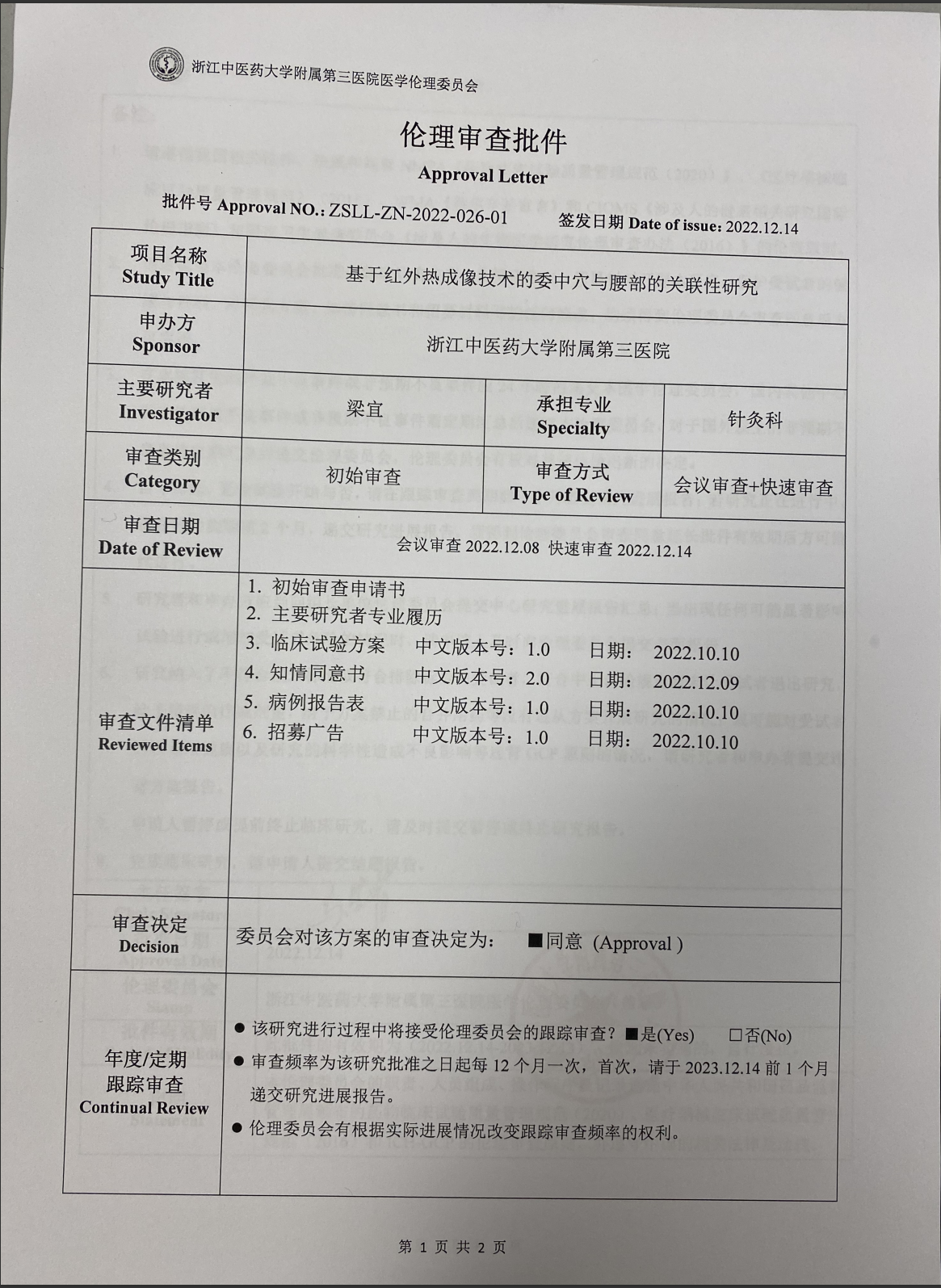


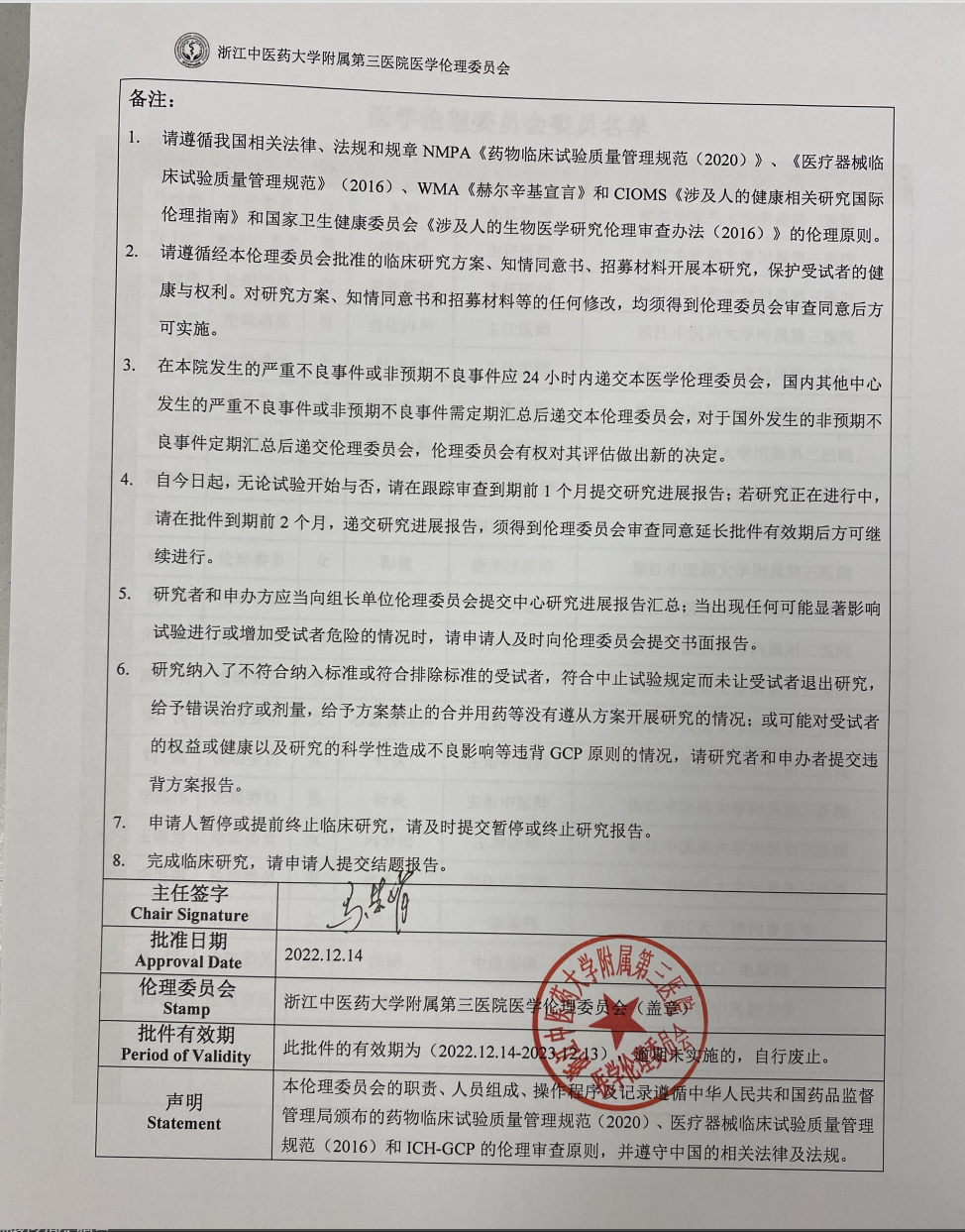


**一项前瞻性、随机、对照临床试验：**

**基于红外热成像技术的委中穴与腰部的关联性研究**

**研究类型：**临床研究

**研究中心：**浙江中医药大学附属第三医院

**本院承担科室：** 针灸科

**本院负责人签字：**

**日期： 年 月**

**本研究将遵循本临床研究方案和GCP实施**

**目录**

[一、研究背景 3](#_Toc116917321)

[二、主要研究内容、目标、方案和进度及拟解决的关键问题： 3](#_Toc116917322)

[1. 研究内容 4](#_Toc116917323)

[2. 研究目的 4](#_Toc116917324)

[3. 研究方案 4](#_Toc116917325)

[3.1. 研究方案 4](#_Toc116917326)

[3.1.1. 样本量的估算 4](#_Toc116917327)

[3.1.2. 随机对照设计和实施 5](#_Toc116917328)

[3.1.3. 纳入标准 6](#_Toc116917329)

[3.1.4. 排除标准 6](#_Toc116917330)

[3.1.5. 剔除和脱落标准 6](#_Toc116917331)

[3.1.6. 中止标准 6](#_Toc116917332)

[3.1.7. 不良事件 7](#_Toc116917333)

[3.2. 干预措施 7](#_Toc116917334)

[3.2.1. 针刺委中穴组： 7](#_Toc116917335)

[3.2.2. 针刺尺泽穴组： 7](#_Toc116917336)

[3.2.3. 艾灸委中穴组： 7](#_Toc116917337)

[3.2.4. 艾灸尺泽穴组： 8](#_Toc116917338)

[3.2.5. 疗效指标和评价 8](#_Toc116917339)

[3.3. 应急处理 8](#_Toc116917340)

[3.4. 技术路线 9](#_Toc116917341)

[三、不良事件（根据课题情况进行描述） 9](#_Toc116917342)

[1. 不良事件定义 9](#_Toc116917343)

[2. 获取不良事件信息 10](#_Toc116917344)

[3. 不良事件的观察与记录 10](#_Toc116917345)

[4. 不良事件的处理 10](#_Toc116917346)

[四、伦理学和质量 10](#_Toc116917347)

[五、数据管理 10](#_Toc116917348)

[六、统计分析 11](#_Toc116917349)

[1. 统计软件 11](#_Toc116917350)

[2. 数据描述 11](#_Toc116917351)

[3. 数据统计 11](#_Toc116917352)

[4. 统计分析计划 11](#_Toc116917353)

[七、最终报告和发表 11](#_Toc116917354)

[八、质量控制 11](#_Toc116917355)

[九、参考文献 12](#_Toc116917356)

# 一、研究背景

针灸作为传统医学的组成之一，因其疗效确切且无毒副作用，在国内外应用广泛，具有普遍较高的接受度，目前已在全球183个国家和地区使用。然而，对于针灸疗效产生的生物学基础及其内在作用机制，至今虽已陆续有高质量论文发表，但仍未得到全面的阐释。随着生命科学研究技术的不断革新，针灸现代研究不断深入，针灸效应规律及其作用机制也被逐步揭开神秘的面纱。如韩济生院士及其团队应用神经生物学技术，研究证实不同频率电针发挥镇痛效应及其起效时间存在差异，其内在生物学基础可能与不同频率电针促不同类型中枢阿片肽在不同时间和不同部位的释放有关^[1-3]^。近日哈佛大学Ma Qiufu教授团队借助于慢病毒技术、基因鼠等生物学技术，揭示了电针刺激效应的区域特异性，发现不同深度和强度电针抗炎效应存在不同的生物学基础，将研究结果发表于《Nature》和《Neuron》^[4,5]^，针灸的国际学术地位也得到了进一步提升。由此可见，推动针灸产生疗效的生物学基础研究无论是对传统针灸的机理阐释还是推动针灸进一步推广发展，都有着举足轻重的作用。

传统的针灸理论是基于前人的临床实践经验不断总结、提炼而成，虽临床疗效稳定、应用广泛，但其客观证据及生物学基础研究仍有所欠缺。因此，在前人经验总结基础上，通过现代科学研究技术，深入揭示针灸经典论述的内在生物学基础显得尤为重要。此外，虽通过动物实验模拟构建人类疾病模型后，可以通过比较各组模型动物的各项生理、生化指标及功能、机能指标来阐释一部分针灸治疗机制；但人的意识形态与社会属性直接导致其与动物之间始终存在着不可逾越的巨大差异，故而基于人体的临床试验研究依旧是不可缺失的一部分。

委中穴治疗腰部疾病的记载，《内经》中就有所提及，《素问·刺腰痛》中记载“足太阳脉令人腰痛，引项脊尻背为重状，刺其郄中太阳正经出血”，而后经诸多医家的归纳总结概述，宋代时已将委中穴主治的基础症状提炼为腰腿部症状^[6]^，后继续归纳概括，便有了《针灸大全》四总穴歌中的“腰背委中求”，沿用至今。临床上常通过在委中穴应用放血疗法^[7]^、电针^[8]^、艾灸^[9]^等手段治疗各种腰部疾患，均获良效。现有研究也初步表明，腰背部与委中穴之间存在着某种客观联系，如刺激委中穴能在腰背部观察到局部血供的丰富^[10]^、局部温度的升高^[11]^、腰部肌肉抗疲劳性的提升^[12]^等改变；当腰背部发生病变时，在委中穴也能观察到力敏^[13]^、热敏^[14]^及局部电阻改变^[15]^等穴位敏化现象。但是委中穴与腰背部之间存在的特异性关联仍有待进一步探索完善。故本研究拟以“腰背委中求”的内涵研究为切入点，通过红外热成像技术，试探求腰部与委中穴的关联性，及不同干预手段对腰部观察区影像的差异性，以期为其科学内涵阐释提供些许参考。

# 二、主要研究内容、目标、方案和进度及拟解决的关键问题：

## 研究内容

选取健康人群为受试者，使用灸行灸法干预，以FLIR E53红外热成像仪为观测仪器，通过检测在不同穴位（委中穴与尺泽穴）施加不同干预（针刺与灸法）后对腰部特定区域范围内的皮温动态变化，以期进一步探究腰部与委中穴存在的关联性，为丰富“腰背委中求”的科学内涵提供客观研究依据。

## 研究目的

通过对不同穴位施加针刺或者艾灸后观测健康受试者腰部局部均温的改变来浅探腰部与委中穴之间存在的关联性及针刺与艾灸对腰部观测区域的影响是否有差异。

## 研究方案

在开始研究前，研究者清楚、口语化地向该受试者解释研究的情况及其潜在风险和益处，获得患者或其授权人同意后，患者或其授权人和研究者在知情同意书上签名和注明日期。患者或其授权人只有在签署了知情同意书后才能进入筛选并继而参加本研究。

### 研究方案

本研究采用随机对照研究设计，严格按照诊断标准、纳入标准和排除标准筛选受试者，确定入组后，按照随机数字表随机分组，直到完成总观察例数后结束试验。研究对象为浙江中医药大学内年龄在18-60岁的健康受试者。

- - 1. 样本量的估算

本项目采用2×2析因设计样本量计算方法，参照先前类似试验中腰部均温改变情况[13,14]及基于健康受试者的预实验结果，做假设如下：

针刺×委中穴组＞灸法×委中穴组＞所有其他组

针刺×委中穴组＞针刺×尺泽穴组

灸法×委中穴组＞灸法×尺泽穴组

基于上述假设预测四组的均温改变值，列表如下：

| Δ（℃）  不同穴位 | 不同干预手段 | |
| --- | --- | --- |
|  | 针刺 | 灸法 |
| 委中穴 | 0.84 | 0.70 |
| 尺泽穴 | 0.34 | 0.20 |

采用双边检验，不考虑交互作用，采用两样本均数比较估算各个组合所需的样本例数（参见孙振球主编的《医学统计学》），具体计算公式如下，选取最大值作为每组的最小样本例数：

$$N=\lambda/{\frac{1}{\sigma^{2}}\sum_{i=1}^{k} \left( \overline{X}_{i}-\bar{X_{0}} \right)^{2}}$$

在α=0.05（双侧）的显著性水平下，选取检验效能（1−β）=80%，根据预实验结果，设定σ=0.26，同时考虑20%的脱失率，最终四组样本总量为140例。

- - 1. 随机对照设计和实施

(1)采用SPSS 20.0软件程序将健康受试者进行随机分配；将180个数字按照1:1:1:1随机分为灸委中穴组，灸尺泽穴，针刺委中穴组，针刺尺泽穴4组，分组信息由项目负责人保存并保密。

(2)编制随机分配卡如下：

| 基于红外热成像技术的委中穴与腰背部的关联性研究 |
| --- |
| 编号： |
| 分组： |
| 干预方法： |

(3)随机分配卡用信封密封，信封上记录与卡片相同的编号。

(4)将内含随机分配卡之信封按编号依次排列

(5)随机分配卡由研究者统一制作、保管，研究时发放给操作者。当合格受试者进入研究时，操作者按其进入的顺序拆开编号相同的信封，根据信封内卡片的规定进行分组，不得做任何更改。参与分组人员不参与数据的统计分析。数据提取统计由不知分组情况的人员负责收集和整理，实行研究者、操作者、统计者三分离。

- - 1. 纳入标准

1. 年龄≥18周岁，性别不限，18.5≤BMI≤23.9 kg/m^2^；
2. 既往身体健康，无器质性疾病；
3. 了解研究过程自愿参加研究并签署知情同意书。
   - 1. 排除标准
4. 女性处于经期、妊娠期或者哺乳期；
5. 无法俯卧完成拍摄者；
6. 测试点有皮肤病或皮肤破损、感觉障碍、瘢痕、赘生物者；
7. 患有严重其他系统疾病，或研究者认为不适合参加研究的；
8. 合并有癫痫、头部损伤或其他相关神经系统疾病者。
   - 1. 剔除和脱落标准

剔除标准（已入组病例但符合以下条件之一者，应予剔除）：（1）在试验中发现不符合纳入、排除标准者；（2）治疗过程中出现明显的不良反应；（3）受试者在入组后未按治疗方案进行治疗。注：剔除的病例应说明，其原始病历应保留备查，不作疗效统计分析，但至少接受一次治疗且有记录者，可参加不良反应分析。

脱落标准（已入组但未完成临床方案的病例，在下列情况应视为脱落）：（1）病人自行退出或失访；（2）治疗过程中出现严重不良反应或不良事件者。

注：脱落的病例应说明原因，其研究病历应保留备查。随访时数据不结转。

- - 1. 中止标准

（1）研究中出现严重不良反应，由专科医生负责评估，确定是否继续或终止研究；

（2）受试者在研究期间出现严重并发症或其他严重疾病，需采取紧急措施者；

（3）受试者有其他原因不能继续本研究者。

- - 1. 不良事件

详细记录不良事件及试验脱落例数及原因。

### 干预措施

本研究将受试者以1:1:1:1随机分为灸委中穴组，灸尺泽穴，针刺委中穴组，针刺尺泽穴4组进行随机对照研究，观察腰部与委中穴间存在的关联性。拟研究时间：2023年 1月 1 日-2023年12月31日。治疗方法具体如下。

- - 1. 针刺委中穴组：

1. 穴位选取：双侧委中穴 (BL40)
2. 定位：参照 2006 年中华人民共和国国家标准（GB/T 12346-2006）《腧穴名称与定位》
3. 针刺操作：俯卧位，穴位常规消毒，针具选用φ25×40mm华佗牌针灸针，在委中穴以75％酒精消毒后，避开神经，血管进行针刺，进针深度约1寸(33mm)左右，行均匀地捻转，捻转角度在90°-180°，频率在60-90次/分，受试者有酸、麻、胀痛等得气感觉时，停止手法操作，留针30 min，留针期间不予手法刺激。
4. 干预时间：30 min
   - 1. 针刺尺泽穴组：
5. 穴位选取：双侧尺泽穴（LU5）
6. 定位：参照 2006 年中华人民共和国国家标准（GB/T 12346-2006）《腧穴名称与定位》
7. 针刺操作：同委中穴操作。
8. 干预时间：30 min
   - 1. 艾灸委中穴组：
9. 穴位选取：双侧委中穴 (BL40)
10. 定位：参照 2006 年中华人民共和国国家标准（GB/T 12346-2006）《腧穴名称与定位》
11. 灸法操作：控制环境温度为25-27℃，湿度40%-60%；受试者取俯卧位，暴露右侧下肢，使用扶阳灸在委中穴区处施加干预
12. 干预时间：30 min
    - 1. 艾灸尺泽穴组：
13. 穴位选取：双侧尺泽穴 (LU5)
14. 定位：参照 2006 年中华人民共和国国家标准（GB/T 12346-2006）《腧穴名称与定位》
15. 灸法操作：同上
16. 干预时间：30 min
    - 1. 疗效指标和评价
17. 基线指标：通过访谈、问卷调查采集受试者性别、年龄、体重、身高、近期腰部出现不适（如腰酸等）频率（周次）、是否接受过针/灸相关治疗等信息，开始干预前用红外热成像仪器记录腰部观测区域（双侧膀胱第二侧线与T12、S1所在水平线围成的几何图形）的平均温度。
18. 主要指标：施加干预30 min后与干预即刻腰部观测区域（双侧膀胱第二侧线与T12、S1所在水平线围成的几何图形）的平均温度变化值
19. 次要指标：
20. 施加干预30min后腰部指定区域的最高温度；
21. 施加干预5 min和15 min后腰部指定区域的平均温度变化值；
22. 委中穴、尺泽穴干预前、干预后30 min局部穴区（φ=2cm）的平均温度；
23. 腰部温热感评估：采用二分类数据（是/否）与NRS评分（0-10）记录受试者在干预期间出现的腰部温热感及其强度。
24. 静息前与干预结束即刻肾俞穴_双_(BL 23)、志室_双_(BL 52)、命门穴(GV 4)及委中穴_双_(BL 40)的痛阈的差异性。
25. 安全性评价：记录不良反应及其表现、发生时间、程度、处理措施、经过、结局等，判断其与治疗的相关性。
26. 依从性评价：对脱落情况及原因进行记录并分析。

### 应急处理

(1)晕针/晕灸：立即停止干预（刺/灸），并将已刺之针全部起出，使患者平卧，头部稍低，解开衣扣，并注意保暖，轻者静卧片刻，即可恢复正常。重者在上述处理基础上，可刺水沟、素髎、内关、足三里、涌泉等穴，亦可灸百会、关元、气海等穴，即可恢复正常。若仍不省人事，呼吸微弱，脉细弱者，采用急救措施并请急诊会诊。

(2)烧烫伤：立即停止干预，予流动冷水冲洗或予冰袋局部冰敷，若未有水泡或水泡较小者与碘伏局部消毒后涂抹烫伤膏；若水泡范围较大，消毒后予无菌注射器挑破水泡，抽净水泡内液体后予无菌敷料包扎。

(3)皮下出血：若微量皮下出血而局部呈小块出血时，不做特殊处理。若局部肿胀疼痛较剧，青紫面积大而且影响到活动功能时，24小时内先予冷敷止血，24小时后予热敷或局部艾灸以促使瘀血消散吸收。

### 技术路线


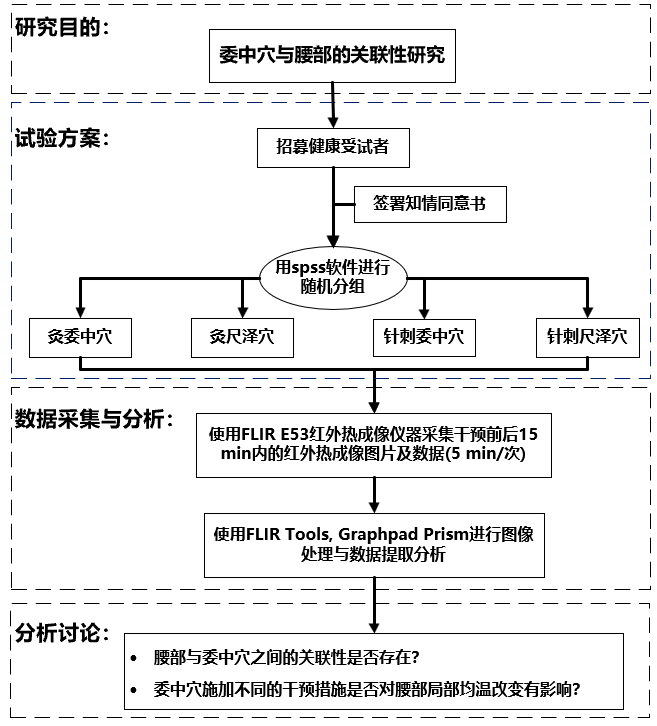


# 三、不良事件（根据课题情况进行描述）

## 不良事件定义

自受试者签署知情同意书后至干预结束，发生与干预措施关联密切的事件，不良事件包括如下：

-- 晕针/晕灸

-- 烧烫伤

-- 皮下出血

## 获取不良事件信息

研究医师通过观察干预期间及干预后受试者穴区的基本情况及受试者自发报告的所有不良事件。

## 不良事件的观察与记录

包括从开始施加干预到干预结束之间发生的任何症状体征与干预手段关联密切的不适症状，如晕针/晕灸，烧烫伤，皮下出血等。

## 不良事件的处理

(1)晕针/晕灸：立即停止干预（刺/灸），并将已刺之针全部起出，使患者平卧，头部稍低，解开衣扣，并注意保暖，轻者静卧片刻，即可恢复正常。重者在上述处理基础上，可刺水沟、素髎、内关、足三里、涌泉等穴，亦可灸百会、关元、气海等穴，即可恢复正常。若仍不省人事，呼吸微弱，脉细弱者，采用急救措施并请急诊会诊。

(2)烧烫伤：立即停止干预，予流动冷水冲洗或予冰袋局部冰敷，若未有水泡或水泡较小者与碘伏局部消毒后突破烫伤膏；若水泡范围较大，消毒后予无菌注射器挑破水泡，抽净水泡内液体后予无菌敷料包扎。

(3)皮下出血：若微量皮下出血而局部呈小块出血时，不做特殊处理。若局部肿胀疼痛较剧，青紫面积大而且影响到活动功能时，24小时内先予冷敷止血，24小时后予热敷或局部艾灸以促使瘀血消散吸收。

# 四、伦理学和质量

本研究开始前将事先获得伦理委员会批准。

在入选患者之前一定要获得其授权同意使用相关数据。为了保护病人的隐私，病人的姓名首字母缩写将会记录在CRF上。

# 五、数据管理

研究者需根据研究方案要求把收集的数据填入病例报告表并用EXCEL进行数据的采集或记录，数据管理由郑思懿负责，需确保临床试验数据的真实性、完整性和准确性。研究结束时，研究者将向数据管理中心递交所有本研究入选的病人的病例报告表，这些病例报告表应是完整的并署名的。从各研究中心收集的病例报告表数据的一致性将被检查，会对不一致的数据发疑问表，需要研究者来澄清。

# 六、统计分析

## 统计软件

由不参与前期试验的第三方统计者进行数据统计分析，用统计软件（SPSS 26.0）等进行统计分析。

## 数据描述

计量资料以均数±标准差（$\bar{x}\pm s$）、中位数、最大值、最小值、四分位数描述，计数资料以百分率（%）表示。

## 数据统计

本试验所有结果使用SPSS 23.0版本进行分析。所有的统计检验均采用双侧检验，*P*＜0.05将被认为所检验的差别有统计学意义。四组间正态分布连续变量的比较采用方差分析(ANOVA)，非正态分布的比较采用Kruskal-Wallis H检验。无法进行正态性转化则采用秩和检验进行比较。计数资料采用卡方检验进行比较。相关分析采用皮尔逊相关分析。采用有序logistics回归分析分析一般资料对最终数据的影响。析因试验主要研究两种处理的主要效应及其相互作用。因此，采用析因设计的重复测量方差分析不同疗法与不同干预穴区对观测区域的影响差异，组间比较采用Sidak检验。

## 统计分析计划

由专业统计人员完成。在所有数据录入、审核完毕后，统计人员应及时完成统计分析工作，并出具书面统计分析报告。

# 七、最终报告和发表

研究结束后，研究报告中将包括研究目的描述、研究中所使用的方法以及结果和结论。

# 八、质量控制

(1) 由课题组制定统一的检测SOP。

(2) 临床试验正式启动前一个月课题组举行专门的培训会，对所有参与课题的研究者进行统一培训。主要对课题实施方案及各项标准操作规程(SOP)进行重点培训，使每个临床研究人员熟悉掌握研究过程和具体实施细则，保证临床研究结论的可靠性。

(3) 临床研究中所有观察结果都应加以核实，反复确认，以保证数据的可靠性、原始性，确保临床研究中的各项结果及结论均来源于原始数据。

(4) 采用专门人员进行试验数据的收集及统计，以控制试验偏倚。委托专业的数据管理公司进行临床数据管理。

(5) 严格实施每月1次的临床研究质量检查。

# 九、参考文献

[1] Han J S. Acupuncture and endorphins[J]. Neurosci Lett, 2004, 361(1-3): 258-61.

[2] Xiang X H, Chen Y M, Zhang J M, et al. Low- and high-frequency transcutaneous electrical acupoint stimulation induces different effects on cerebral μ-opioid receptor availability in rhesus monkeys[J]. J Neurosci Res, 2014, 92(5): 555-63.

[3] Huo R, Han S P, Liu F Y, et al. Responses of Primary Afferent Fibers to Acupuncture-Like Peripheral Stimulation at Different Frequencies: Characterization by Single-Unit Recording in Rats[J]. Neurosci Bull, 2020, 36(8): 907-918.

[4] Liu S, Wang Z, Su Y, et al. A neuroanatomical basis for electroacupuncture to drive the vagal-adrenal axis[J]. Nature, 2021, 598(7882): 641-645.

[5] Liu S, Wang Z F, Su Y S, et al. Somatotopic Organization and Intensity Dependence in Driving Distinct NPY-Expressing Sympathetic Pathways by Electroacupuncture[J]. Neuron, 2020, 108(3): 436-450.e7.

[6] 黄龙祥. 腧穴主治的规范化表述. 中国针灸, 2007: 823-827.

[7] 周娜. 委中穴放血干预不同证型腰痛的临床疗效观察[D]. 江西中医药大学, 2020.

[8] 史景. 电针委中穴对下腰痛患者表面肌电特征的影响[D]. 2018.

[9] 覃斯妤, 焦琳, 程攀, et al. 基于数据挖掘的艾灸治疗腰痹病临床应用规律分析. 针灸临床杂志, 2021: 75-80.

[10] 范伊凡. 针刺单侧委中穴对正常人腰骶部温度变化的研究[D]. 2015.

[11] 王苓苓, 张维波, 谢衡辉, et al. 使用血流成像技术对"腰背委中求"经典理论的验证. 针刺研究, 2007: 247-251.

[12] 白硕. 电针委中穴缓解腰背肌疲劳的表面肌电研究[D]. 北京中医药大学, 2017.

[13] 应文强, 彭桂秀, 成锐, et al. 腰椎间盘突出患者力敏腧穴分布规律研究. 江西中医药, 2021: 48-51.

[14] 李伟, 安鑫. 腰椎间盘突出症腧穴热敏化红外客观显示研究. 江西中医学院学报, 2010: 24-26.

[15] 宋佳杉, 吴晓林, 佘延芬, et al. 膀胱经、胆经特定穴皮肤电阻对腰椎间盘突出症患者的反应研究. 针灸临床杂志, 2018: 30-34.

[16] 陈冬荔, 肖瑶, 邹德辉, et al. 针刺委中穴对轻中度腰痛局部温度变化的影响[J]. 世界中医药, 2018, 13(06): 1529-1532.
