# Supplementary material for: Effect of acupuncture or moxibustion at Acupoints Weizhong (BL40) or Chize (LU5) on the change in lumbar temperature in healthy adults: A study protocol for a randomized controlled trial with a 2 × 2 factorial design
Source: PLoS One. 2023 Oct 30;18(10):e0291536. doi: 10.1371/journal.pone.0291536 (PMC10615297; doi:10.1371/journal.pone.0291536)
Supplement: S2 File — (DOCX) [file pone.0291536.s003.docx]

**A prospective, randomized, controlled clinical trial:**

**The association between Weizhong point and waist based on infrared thermal imaging technology**

| **Study type:** | Clinical study |
| --- | --- |
| **Study Center:** | The Third Affiliated Hospital of Zhejiang Chinese Medical University |
| **Department:** | Acupuncture department |
| **Signature:** | Yi Liang |

Date: 2022.10.10

The study will be conducted in accordance with this clinical study protocol and GCP

Contents

[I. Research Background 3](#_Toc116917321)

[Ii. Main research content, objectives, program and progress, and key problems to be solved: 3](#_Toc116917322)

[1. Research content 4](#_Toc116917323)

[2. Objectives of the study 4](#_Toc116917324)

[3. Study protocol 4](#_Toc116917325)

[3.1. Study Protocol 4](#_Toc116917326)

[3.1.1. Estimation of sample size 4](#_Toc116917327)

[3.1.2. Randomized control design and conduct 5](#_Toc116917328)

[3.1.3. Inclusion criteria 6](#_Toc116917329)

[3.1.4. Exclusion criteria 6](#_Toc116917330)

[3.1.5. Exclusion and drop-out criteria 6](#_Toc116917331)

[3.1.6. Termination criteria 6](#_Toc116917332)

[3.1.7. Adverse events 7](#_Toc116917333)

[3.2. Interventions 7](#_Toc116917334)

[3.2.1. Acupuncture at Weizhong Point Group: 7](#_Toc116917335)

[3.2.2. Acupuncture Chize acupoint group: 7](#_Toc116917336)

[3.2.3. Moxibustion at Weizhong Point Group: 7](#_Toc116917337)

[3.2.4. Moxibustion at Chize point group: 8](#_Toc116917338)

[3.2.5. Therapeutic indexes and evaluation 8](#_Toc116917339)

[3.3. Emergency treatment 8](#_Toc116917340)

[3.4. Technical route 9](#_Toc116917341)

[Iii. Adverse events (described according to the subject) 9](#_Toc116917342)

[1. Definition of adverse events 9](#_Toc116917343)

[2. Obtain information on adverse events 10](#_Toc116917344)

[3. Observation and recording of adverse events 10](#_Toc116917345)

[4. Management of adverse events 10](#_Toc116917346)

[4. Ethics and quality 10](#_Toc116917347)

[V. Data management 10](#_Toc116917348)

[6. Statistical analysis 11](#_Toc116917349)

[1. Statistical software 11](#_Toc116917350)

[2. Data description 11](#_Toc116917351)

[3. Data statistics 11](#_Toc116917352)

[4. Statistical analysis plan 11](#_Toc116917353)

[Vii. Final report and publication 11](#_Toc116917354)

[Viii. Quality control 11](#_Toc116917355)

[Ix. References 12](#_Toc116917356)

# I. Research Background

As one of the components of traditional medicine, acupuncture and moxibustion is widely used at home and abroad due to its exact curative effect and no toxic side effects. It has been widely accepted and used in 183 countries and regions around the world. However, the biological basis and intrinsic mechanism of acupuncture and moxibustion have not been fully explained, although high-quality papers have been published in succession. With the continuous innovation of life science research technology and the deepening of modern research on acupuncture and moxibustion, the mysterious veil of the effect law and mechanism of acupuncture and moxibustion has been gradually unveiled. For example, academician Han Jisheng and his team have applied neurobiological techniques to prove that there are differences in the analgesic effect and onset time of different frequencies of electroacupuncture. The intrinsic biological basis may be related^[1-3]^ to the release of different types of central opioid peptides at different times and different parts promoted by different frequencies of electroacupuncture. Recently, Professor Ma Qiufu's team at Harvard University revealed the regional specificity of electroacupuncture stimulation by using biological technologies such as lentivirus technology and gene mice, and found that there were different biological bases for the anti-inflammatory effects of electroacupuncture at different depths and intensities. The research results were published in Nature and Neuron^[4,5]^. The international academic status of acupuncture has also been further improved. Therefore, the biological basic research of acupuncture and moxibustion plays a pivotal role in both the explanation of the mechanism of traditional acupuncture and moxibustion and the promotion of its further development.

The traditional theory of acupuncture and moxibustion is continuously summarized and refined based on the clinical practice experience of predecessors. Although the clinical efficacy is stable and widely used, there is still a lack of objective evidence and biological basic research. Therefore, on the basis of the summary of previous experience, it is particularly important to deeply reveal the intrinsic biological basis of the classic discussion of acupuncture and moxibustion through modern scientific research technology. In addition, although the human disease model is constructed through animal experiment simulation, a part of the therapeutic mechanism of acupuncture and moxibustion can be explained by comparing the physiological and biochemical indexes and functional indexes of the model animals in each group. However, the ideology and social attributes of human beings directly lead to insurable huge differences between human beings and animals. Therefore, clinical trials based on human beings are still an indispensable part.

It is mentioned in Neijing (The Classic of Internal Medicine) that Weizhong acupoint is used to treat waist diseases. It is recorded in Plain Questions: "The foot Taiyang pulse causes low back pain, citing the back of the neck ridge as the severe form, and needling it at Zhongtaiyang for severe bleeding". After the summary and summary of many doctors, the basic symptoms of Weizhong acupoint were refined as waist and leg^[6]^ symptoms in the Song Dynasty, and then continued to summarize and summarize. Then, the "Wei zhong qiu" in the song of the four general acupoints in the Complete Compendium of Acupuncture and Moxibustion, which is still in use today. In clinical practice, bloodlet therapy^[7]^, electroacupuncture^[8]^, mo^[9]^xibustion and other methods are often applied at Weizhong point to treat various lumbar disorders, and good effects are obtained. The existing studies have preliminarily shown that there is some objective relationship between the waist and back and Weizhong point. For example, the stimulation of Weizhong point can observe the rich^[10]^ local blood supply, the increase^[11]^ of local temperature, and the improvement^[12]^ of waist muscle fatigue resistance. When the waist and back lesions occur, acupoint sensi^[13]^tization phenomena such as force sensitivity, heat^[14]^ sensitivity and local electrical resistance changes^[15]^ can also be observed at Weizhong point. However, the specific association between Weizhong point and waist and back still needs to be further explored and improved. Therefore, this study intends to take the connotation study of "Weizhong acupoint on waist and back" as the entry point, to explore the correlation between the waist and Weizhong acupoint through infrared thermal imaging technology, and to explore the differences in the image of the waist observation area by different intervention methods, in order to provide some reference for the interpretation of its scientific connotation.

# Ii. Main research content, objectives, program and progress, and key problems to be solved:

## Research Content

The healthy people were selected as subjects and treated with moxibustion. FLIR E53 infrared thermal imager was used as the observation instrument to detect the dynamic changes of skin temperature in a specific area of the waist after different interventions (acupuncture and moxibustion) at different acupoints (Weizhong point and Chize point), in order to further explore the relationship between the waist and Weizhong point. To provide an objective research basis for enriching the scientific connotation of "waist and back".

## Objectives of the study

To explore the relationship between the waist and Weizhong points and whether acupuncture and moxibustion have different effects on the observation area of waist by observing the changes of local average temperature of waist in healthy subjects after applying acupuncture or moxibustion to different points.

## Study PROTOCOL

The investigator clearly and orally explained the study and its potential risks and benefits to the subject before starting the study. After obtaining consent from the patient or his or her authorized person, the patient or his or her authorized person and the investigator signed and dated the informed consent form. Patients or their authorized persons could be screened for participation in the study only if they had provided written informed consent.

### Study Protocol

Subjects were screened strictly according to the diagnostic criteria, inclusion criteria and exclusion criteria. After confirmation of enrollment, they were randomly divided into groups according to the random number table until the total number of observation was completed. Healthy subjects aged 18-40 years old from Zhejiang Chinese Medical University were included in this study.

- - 1. Estimation of sample size

This project uses the sample size calculation method of 2×2 factorial design. Referring to the changes^[10,16]^ of waist average temperature in previous similar experiments and the results of pre-experiments, the following assumptions are made:

- Acupuncture × Weizhong acupoint (right) group > moxibustion × Weizhong acupoint (right) group > all other groups
- Acupuncture × Weizhong (right) group > acupuncture × Chize (right) group
- Moxibustion method × Weizhong point (right) group > Moxibustion method × Chize point (right) group

| Δ (℃)  Different points | Different interventions | |
| --- | --- | --- |
|  | Acupuncture | Moxibustion |
| Weizhong acupoint (right) | 0.84 | 0.7 |
| Chieze Cave (right) | 0.34 | 0.2 |

Two-sided test was used without considering the interaction effect, and the number of samples required for each combination was estimated by comparing the means of two samples (see Medical Statistics edited by Sun Zhenqiu). The specific calculation formula was as follows, and the maximum value was selected as the minimum number of samples in each group:

$$N=\lambda/{\frac{1}{\sigma^{2}}\sum_{i=1}^{k} \left( \overline{X}_{i}-\bar{X_{0}} \right)^{2}}$$

Where N is the required amount of samples; n_1_ is the sample_2_ size required for comparison between the acupuncture and moxibustion at the Weizhong acupoint group, the acupuncture and moxibustion at the Chize acupoint group,_3_ the acupuncture and moxibustion at_4_ the Chize acupoint group, and the acupuncture and moxibustion at the Chize acupoint group. Because n_4_ is not the key object of this study, it is not included in the calculation of sample size. δ=µ_1_−µ,_2_ is the difference between the two population means,σ is the population standard deviation (assuming the two population standard deviation is equal); u_α_ and u_β_ are the values of u corresponding to the test level α and the type I error probability β, respectively.

At the significance level of α=0.05 (bilateral), the test efficacy (1−β) =80% was selected, and σ=0.26 was set according to the pre-experimental results, and the loss rate of 20% was considered. The final total number of samples in the four groups was 140.

- - 1. A randomized controlled trial was designed and conducted

(1) Healthy subjects were randomly assigned by SPSS 20.0 software program. The 180 digits were randomly divided into a moxibustion group at Weizhong point, a moxibustion group at Chize point, an acupuncture group at Weizhong point and an acupuncture group at Chize point according to the ratio of 1:1:1:1. The grouping information was kept confidential by the project leader.

(2) The random allocation card was prepared as follows:

| Association between Weizhong point and waist and back based on infrared thermal imaging technology |
| --- |
| No. : |
| Groups: |
| Methods of intervention: |

(3) The random assignment card is sealed in an envelope with the same number recorded on the envelope as the card.

(4) Arrange the envelopes containing the randomization cards in order of number

(5) The randomization cards were made and kept by the investigators and distributed to the operators during the study. When eligible subjects entered the study, the operator opened the same numbered envelopes in the order in which they entered and grouped according to the rules of the cards inside the envelopes without making any changes. Participants in the group assignment did not participate in the statistical analysis of the data. Data extraction and statistics were collected and sorted by the personnel who did not know the grouping, and the researchers, operators, and statisticians were separated.

- - 1. Inclusion criteria

1. Age ≥18 years old, both sexes, 18.5≤BMI≤23.9 kg/m^2^;
2. Previous good health without organic diseases;
3. Understand the study process voluntarily participated in the study and signed the informed consent form.
   - 1. Exclusion Criteria
4. Women who are menstruating, pregnant, or breastfeeding;
5. Unable to complete the filming in a prone position;
6. Those with skin diseases or skin damage, sensory disorders, scars or vegetations at the test points;
7. Patients with serious systemic diseases, or those considered by the investigators to be unsuitable for the study;
8. Patients with epilepsy, head injury or other related neurological diseases.
   - 1. Exclusion and expulsion criteria

Exclusion criteria (those who have been enrolled but meet one of the following criteria should be excluded) : (1) Those who do not meet the inclusion and exclusion criteria are found in the trial; (2) obvious adverse reactions occurred during the treatment; (3) The subjects were not treated according to the treatment plan after enrollment. Note: The excluded cases should be explained, and the original medical records should be kept for future reference. No statistical analysis of efficacy will be performed, but those who have received at least one treatment and have records can participate in the analysis of adverse reactions.

Drop-out criteria (patients who had been enrolled but had not completed the clinical protocol were considered drop-out in the following circumstances) : (1) patients withdrew or lost follow-up; (2) Serious adverse reactions or adverse events occurred during treatment.

Note: The reasons for dropouts should be explained, and the study medical records should be kept for future reference. Data were not carried forward at follow-up.

- - 1. Termination criterion

(1) Specialists will be responsible for the evaluation of severe adverse reactions during the study to determine whether to continue or terminate the study;

(2) if the subjects developed serious complications or other serious diseases during the study period and needed to take emergency measures;

(3) the subjects could not continue the study for other reasons.

- - 1. Adverse events

Adverse events and the reasons for dropouts were recorded in detail.

### Interventions

In this study, the subjects were randomly divided into a moxibustion group at Weizhong point, a moxibustion group at Chize point, an acupuncture group at Weizhong point, and an acupuncture group at Chize point at a ratio of 1:1:1:1 for a randomized controlled trial to observe the relationship between waist and Weizhong point. Proposed research time: January 1, 2023 to December 31, 2023. Specific treatments are as follows.

- - 1. Acupuncture at Weizhong acupoint group:

1. Acupoint selection: Bilateral Weizhong (BL40)
2. Location: Refer to the national standard of the People's Republic of China (GB/T 12346-2006) "Acupoint Name and Location"
3. Acupuncture operation: The acupuncture needles of φ25×40mm Huatuo brand were selected and sterilized at Weizhong points with 75% alcohol. Acupuncture was performed avoiding the nerves and blood vessels. The depth of the needles was about 1 cun (33mm). When the subject had deqi sensation such as acid, numbness, and swelling pain, the manipulation was stopped, and the needle was retained for 30 minutes. No manipulation stimulation was given during the retention period.
4. The intervention time was 30 min
   - 1. Acupuncture Chize acupoint group:
5. Acupoint selection: Bilateral Chize (LU5)
6. Location: Refer to the national standard of the People's Republic of China (GB/T 12346-2006) "Acupoint Name and Location"
7. Acupuncture operation: the same as Weizhong point operation.
8. The intervention time was 30 minutes
   - 1. Moxibustion committee point group:
9. Acupoint selection: bilateral Weizhong (BL40)
10. Location: Refer to the national standard of the People's Republic of China (GB/T 12346-2006) "Acupoint Name and Location"
11. Moxibustion operation: the ambient temperature was controlled at 25-27℃, and the humidity was controlled at 40%-60%. The subjects were placed in the prone position, with their right lower limb exposed, and intervention was applied to the Weizhong acupoint area with Fuyang moxibustion
12. The intervention time was 30 min
    - 1. Moxibustion at Chize point group:
13. Acupoint selection: Bilateral Chize (LU5)
14. Location: Refer to the national standard of the People's Republic of China (GB/T 12346-2006) "Acupoint Name and Location" in 2006.
15. Moxibustion method operation: same as above
16. The intervention time was 30 minutes
    - 1. Efficacy indicators and evaluation
17. Baseline MEASURES: Through interviews and questionnaires, the subjects' gender, age, weight, height, frequency (weekly) of waist discomfort (such as waist acid, etc.), and whether they had received acupuncture/moxibustion related treatment were collected. Before the intervention, the average temperature of waist observation area (the geometric figure surrounding the second lateral line of bilateral bladder and the horizontal lines of T12 and S1) was recorded by infrared thermal imaging instrument.
18. The main outcome measures were the average temperature changes in the waist observation area (the geometric figure surrounded by the second lateral line of bilateral bladder and the horizontal lines T12 and S1) 30 minutes after the intervention and immediately after the intervention
19. Secondary outcome measures:
20. The highest temperature in the designated area of the waist 30min after applying the intervention;
21. The average temperature change value of the designated area of the waist was measured 5 min and 15 min after intervention.
22. The average temperature of local acupoints (φ=2cm) at Weizhong and Chize before intervention and 30 min after intervention were recorded.
23. Evaluation of waist warm sensation: Binary data (yes/no) and NRS score (0-10) were used to record the waist warm sensation and its intensity during the intervention.
24. The differences of pain threshold before rest and immediately after the intervention were compared at Shen_双_shu (BL 23), Zhishi_双_ (BL 52), Mingmen (GV 4) and We_双_izhong (BL 40).
25. Safety evaluation: the adverse reactions and their manifestations, occurrence time, degree, treatment measures, process and outcome were recorded, and the correlation between the adverse reactions and the treatment was judged.
26. Compliance evaluation: the drop-out situation and reasons were recorded and analyzed.

### Emergency response

(1) Dizzy acupuncture/moxibustion: the intervention (acupuncture/moxibustion) should be stopped immediately, all the acupuncture needles should be removed, the patient should lie flat with the head slightly lower, unbutton his clothes, and pay attention to keep warm. If he lies still for a while, he can return to normal. On the basis of the above treatment, the severe cases can be treated with acupuncture at Shuigou (GV 26), Ciliao (BL 32), Neiguan (PC 6), Zusanli (ST 36), Yongquan (SP 9), etc., or moxibustion at Baihui (GV 20), Guanyuan (CV 4), Qihai (CV 6), etc. If the patient is still unconscious, breathing is weak, and the pulse is weak, first aid measures should be taken and emergency consultation should be requested.

(2) Burn injury: the intervention was stopped immediately, and the patients were rinsed with running cold water or applied with ice packs locally. If there were no blisters or small blisters, topical scald ointment was applied after disinfection with iodophor. If the scope of blisters is large, after disinfection, the blisters are broken by sterile syringes, and the liquid inside the blisters is drained, and then the sterile dressing is applied.

(3) Subcutaneous hemorrhage: if there is a small amount of subcutaneous hemorrhage and local bleeding, no special treatment will be done. If the local swelling and pain is severe, the area of cyan is large and affects the activity function, cold compress is given within 24 hours to stop bleeding, and hot compress or local moxibustion is given after 24 hours to promote the stasis to dissipate and absorb.

### Technical route

**
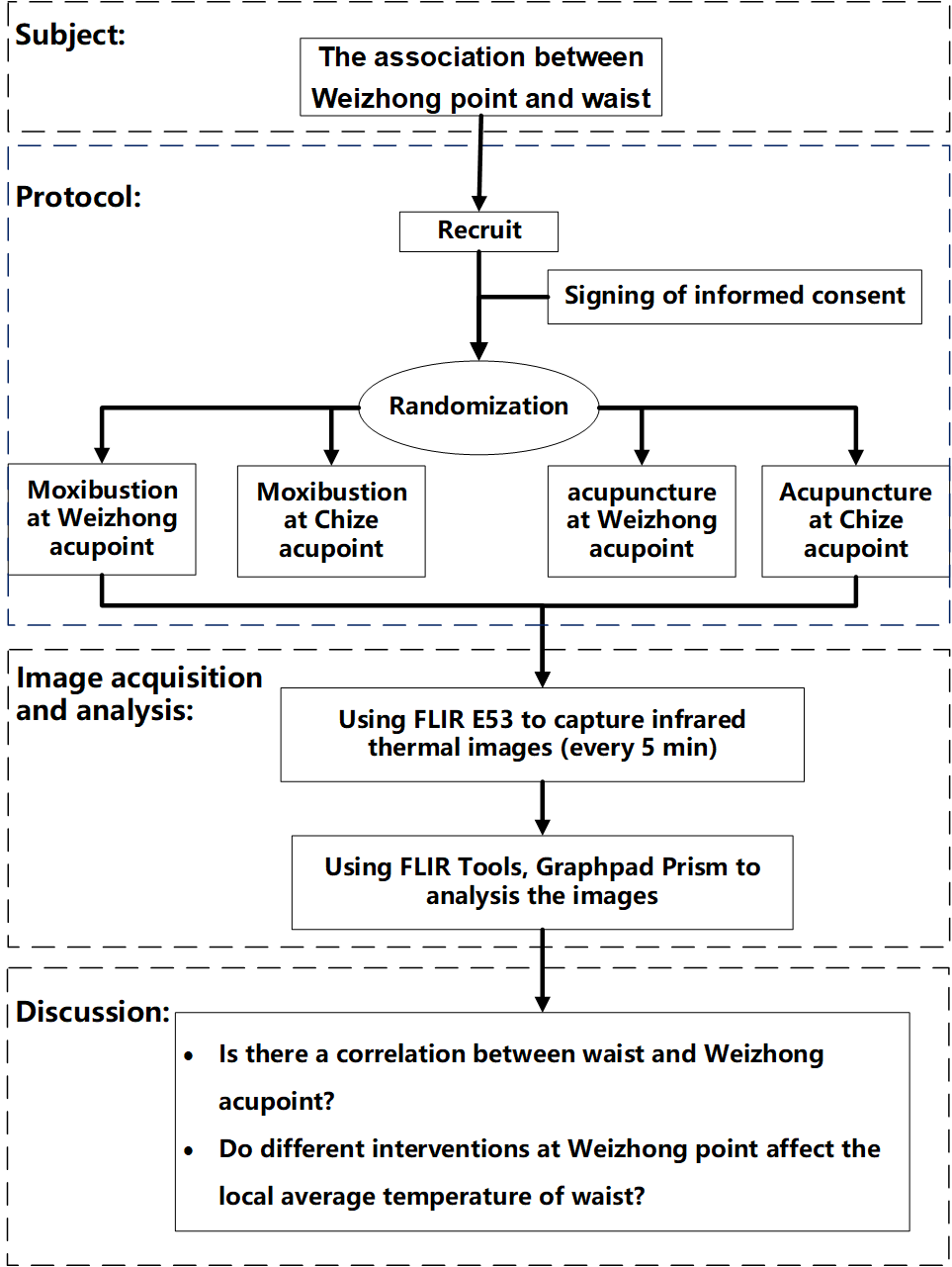
**

# Iii. Adverse events (described according to the subject)

## Definition of adverse events

From the time the subject signed the informed consent to the end of the intervention, events closely related to the intervention occurred, including the following:

-- Acupuncture/dizzy moxibustion

-- Burn and scald

-- Subcutaneous bleeding

## Information on adverse events was obtained

The basic information of the acupoint area and all adverse events reported by the subjects during and after the intervention were observed by the research doctors.

## Adverse events were observed and recorded

Any symptoms and signs that occurred from the beginning of the intervention to the end of the intervention were closely related to the intervention method, such as dizzy/dizzy moxibustion, burn and scald, subcutaneous hemorrhage, etc.

## Management of adverse EVENTS

(1) Dizzy acupuncture/dizzy moxibustion: stop the intervention (acupuncture/moxibustion) immediately, and remove all the acupuncture needles, make the patient lie flat with the head slightly lower, unbutton his clothes, and pay attention to keep warm. If he lies still for a while, he can return to normal. On the basis of the above treatment, the severe cases can be treated with acupuncture at Shuigou (GV 26), Ciliao (BL 32), Neiguan (PC 6), Zusanli (ST 36), Yongquan (SP 9), etc., or moxibustion at Baihui (GV 20), Guanyuan (CV 4), Qihai (CV 6), etc. If the patient is still unconscious, breathing is weak, and the pulse is weak, first aid measures should be taken and emergency consultation should be requested.

(2) Burn injury: the intervention was stopped immediately, and the patients were rinsed with running cold water or applied with ice packs locally. If there were no blisters or small blisters, the patients were locally sterilized with iodarone and then broke through the burn ointment. If the range of blisters was large, sterile syringes were used to break the blisters after disinfection, and sterile dressings were used to bandage the blisters after removing the liquid in the blisters.

(3) Subcutaneous hemorrhage: if there is a small amount of subcutaneous hemorrhage and local bleeding, no special treatment will be done. If the local swelling and pain is severe, the area of cyan is large and affects the activity function, cold compress is given within 24 hours to stop bleeding, and hot compress or local moxibustion is given after 24 hours to promote the stasis to dissipate and absorb.

# 4. Ethics and quality

Prior ethics committee approval will be obtained before the start of this study.

It was important to obtain authorization for the use of the data before enrollment. To protect patient privacy, patient initials will be recorded on the CRF.

# 5. Data management

Investigators were required to fill in the collected data into the case report form and collect or record the data in EXCEL according to the requirements of the study protocol. Zheng Siyi was responsible for data management to ensure the authenticity, completeness and accuracy of the clinical trial data. At the end of the study, case report forms of all enrolled patients will be submitted to the data management center by the investigator. These case report forms should be completed and signed. Case-report form data collected from the sites will be checked for consistency, and question forms will be issued for inconsistent data, requiring investigator clarification.

# 6. Statistical analysis

## Statistical software

The data were analyzed by a third party statistician who was not involved in the preliminary study. Statistical software (SPSS 26.0) was used for statistical analysis.

## Description of data

Measurement data were described by mean ± standard deviation (), median, maximum, minimum and quartile, and enumeration data were described by percentage (%).$\bar{x}\pm s$

## Statistics of data

All the results were analyzed with the use of SPSS, version 23.0. All statistical tests were two-sided, and a *P* value of less than 0.05 was considered to indicate statistical significance. Normally distributed continuous variables were compared among the four groups using analysis of variance (ANOVA), and non-normally distributed variables were compared using the Kruskal-Wallis H test. If normal transformation could not be performed, the rank-sum test was used for comparison. Count data were compared using the chi-square test. Pearson correlation analysis was used for correlation analysis. Ordered logistics regression analysis was used to analyze the influence of general data on the final data. The factorial test focused on the main effects of the two treatments and their interaction. Therefore, repeated measures ANOVA with factorial design was used to analyze the differences in the effects of different treatments and different intervention acupoint areas on the observation area, and Sidak test was used for comparison between groups.

## Statistical Analysis Plan

Done by professional statisticians. After all data entry and review, the statistician should complete the statistical analysis work in time and issue a written statistical analysis report.

# Vii. Final report and publication

After the study, the study report will include a description of the study objectives, the methods used in the study, and the results and conclusions.

# Viii. Quality control

(1) The test SOP should be formulated by the research group.

(2) A special training meeting was held one month before the official start of the clinical trial, and unified training was conducted for all investigators. The implementation plan and standard operating procedures (Sops) were mainly trained to make every clinical researcher familiar with the research process and specific implementation rules, and to ensure the reliability of clinical research conclusions.

(3) All observations in clinical research should be verified and repeatedly confirmed to ensure the reliability and originality of the data, and to ensure that the results and conclusions in clinical research are derived from the original data.

(4) To control the bias of the trial, special personnel should be employed to collect and count the trial data. A professional data management company was commissioned for clinical data management.

(5) The quality inspection of clinical research should be strictly carried out once a month.

# Ix. References

[1] Han J S. Acupuncture and endorphins[J]. Neurosci Lett, 2004, 361(1-3): 258-61.

[2] Xiang X H, Chen Y M, Zhang J M, et al. Low- and high-frequency transcutaneous electrical acupoint stimulation induces different effects on cerebral μ-opioid receptor availability in rhesus monkeys[J]. J Neurosci Res, 2014, 92(5): 555-63.

[3] Huo R, Han S P, Liu F Y, et al. Responses of Primary Afferent Fibers to Acupuncture-Like Peripheral Stimulation at Different Frequencies: Characterization by Single-Unit Recording in Rats[J]. Neurosci Bull, 2020, 36(8): 907-918.

[4] Liu S, Wang Z, Su Y, A neuroanatomical basis for electroacupuncture to drive the vagal adrenal axis[J]. Nature, 2021, 598(7882): 641-645.

[5] Liu S, Wang Z F, Su Y S, et al. Somatotopic Organization and Intensity Dependence in Driving Distinct NPY-Expressing Sympathetic Pathways by Electroacupuncture[J]. Neuron, 2020, 108(3): 436-450.e7.

[6] Huang Longxiang. Standardized expression of indications of acupoints. Chinese Acupuncture and Moxibustion, 2007: 823-827.

[7] ZHOU Na. Clinical observation of Weizhong point bloodletting intervention on different syndrome types of low back pain [D]. Jiangxi University of Traditional Chinese Medicine, 2020.

[8] SHI Jing. Effect of electroacupuncture at Weizhong point on surface electromyography characteristics in patients with low back pain [D]. 2018.

[9] Tan S Y, Jiao L, Cheng P, et al. Analysis of clinical application rules of moxibustion in the treatment of lumbar arthralgia based on data mining. Clinical Journal of Acupuncture and Moxibustion, 2021: 75-80.

[10] Fan Yifan. Effect of acupuncture on temperature change of lumbosacral region in normal people [D]. 2015. (in Chinese)

[11] Wang L L, Zhang W B, Xie H H, et al. Using blood flow imaging technology to verify the classic theory of "waist and back". Acupuncture Research, 2007:247-251.

[12] Bai Shuo. Surface electromyography study of electroacupuncture at Weizhong point in relieving lumbar and back muscle fatigue [D]. Beijing University of Chinese Medicine, 2017.

[13] Ying W Q, Peng G X, Cheng R, et al. Study on the distribution of force sensitive acupoints in patients with lumbar disc herniation. Jiangxi Traditional Chinese Medicine, 2021: 48-51.

[14] Li Wei, An Xin. Research on the objective display of heat-sensitive infrared in acupoints of lumbar disc herniation. Journal of Jiangxi University of Traditional Chinese Medicine, 2010: 24-26.

[15] Song J S, Wu X L, She Y F, et al. Skin resistance of specific points of bladder meridian and bile meridian in patients with lumbar disc herniation. Clinical Journal of Acupuncture and Moxibustion, 2018: 30-34.

[16] Chen D L, XIAO Y, Zou D H, et al. Effect of acupuncture at Weizhong point on local temperature change in mild to moderate low back pain [J]. World Traditional Chinese Medicine, 2018, 13(06): 1529-1532.
