# Supplementary material for: Effect of acupuncture or moxibustion at Acupoints Weizhong (BL40) or Chize (LU5) on the change in lumbar temperature in healthy adults: A study protocol for a randomized controlled trial with a 2 × 2 factorial design
Source: PLoS One. 2023 Oct 30;18(10):e0291536. doi: 10.1371/journal.pone.0291536 (PMC10615297; doi:10.1371/journal.pone.0291536)
Supplement: Attachment — Submitted filename: Response to Reviewers.docx [file pone.0291536.s005.docx]

Dear editor Boram Lee and reviewers,

Thank you very much for giving us an opportunity to revise our manuscript. We appreciate the editor and reviewers very much for their positive and constructive comments and suggestions regarding our manuscript “Effect of acupuncture or moxibustion at Acupoints Weizhong or Chize on the change in lumbar temperature in healthy adults: a study protocol for a randomized controlled trial with a 2 × 2 factorial design (PONE-D-23-12555)”. Your insightful comments and suggestions have significantly contributed to improving the quality and clarity of our work. We have carefully considered each of your comments and have made the necessary revisions accordingly. Here are our responses:

**Response to Additional Editor Comments:**

***English must be improved before publishing, and authors are encouraged to submit English editorial certificate.***

***Response:*** Thank you for your suggestion. The manuscript has been revised to enhance its English quality, and we have included the English editorial certificate as a PDF attachment in the submission.

**Reply to Reviewers' comments:**

***Reviewer #1: The authors have adjusted the text, the revisions have strengthened the clarity and methodology. The protocol technically sound and will lead to a meaningful outcome. It can provide evidence of relationship between BL 40 and "waist and back".***

***Response:*** Thank you for your approval.

***Reviewer #3:***

***The authors have responded constructively to all of the review comments.***

***It would be best if the authors exactly specify the statistical analysis of the primary endpoint. The analysis of variance is a rather general term for the overall methodology. The authors should specify what exactly they will test. One possibility is to test for any effect (inclusive of main effect and/or interaction effect). This could be performed via an omnibus test of the full model versus a restricted model that does not include treatment (or any interaction involving treatment).***

***Response:*** Thank you for your professional advice. We have operationalized the statistical approach as Two-Way analysis of variance, and we have refined the statistical strategy accordingly. we have revised the manuscript as following: “For the primary outcome (mean temperature in the lumbar region at the last minute of the intervention period), two-way analysis of variance (two-way ANOVA) will be used with a full factorial model that includes the main effect of acupoints and interventions and the interaction effect. If there is no interaction effect, simple effects of acupoints and interventions will be analyzed. The model will be adjusted by including baseline temperature as a covariate.”

We are confident that the revised manuscript now meets the high standards set by the journal. We believe that these revisions have strengthened the clarity, methodology, and overall contribution of our study. We hope that you will find our responses and revisions satisfactory.

Once again, we sincerely appreciate your thorough review and constructive feedback. We would like to express our gratitude for your time and expertise in evaluating our work. We believe that your inputs have significantly improved the quality of our manuscript.

We kindly request that you reconsider our manuscript for publication in Plos one. We are confident that the revised version now meets all the requirements and would make a valuable contribution to the scientific community.

Thank you for your attention and consideration.

Sincerely,

Yi Liang
